# Total eclipse in the heart

**DOI:** 10.1007/s12471-017-1058-0

**Published:** 2017-12-14

**Authors:** R. J. de Winter

**Affiliations:** Department of Cardiology, Academic Medical Center, Amsterdam, The Netherlands

What is the diagnosis of the condition treated in this angiogram (Fig. 1)?

**Fig. 1:**
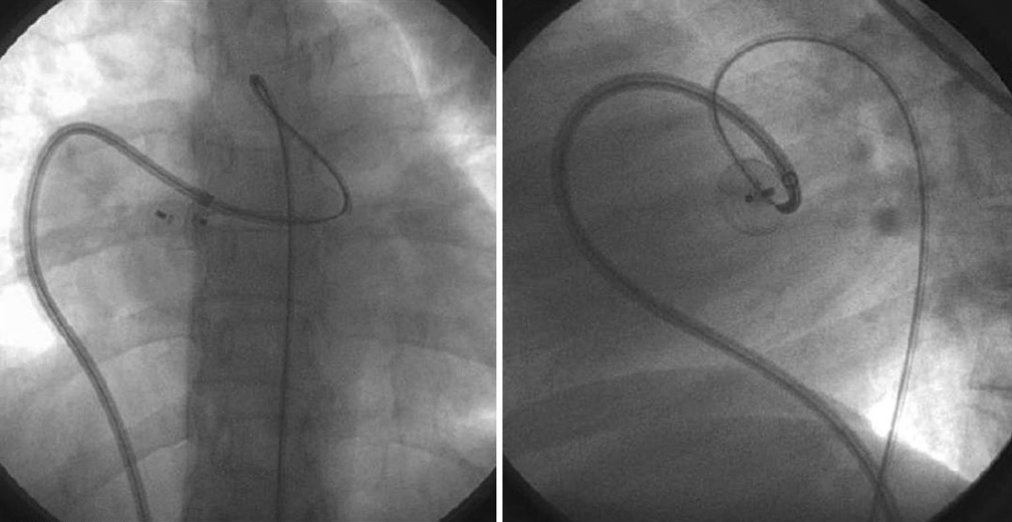
Image puzzle nr 1, simultaneous orthogonal views

*For this new section, authors are invited to submit images of cardiovascular conditions that can be of value to our readers because the images are interesting, intriguing, rare and illustrative, thought-provoking, relevant to recent discussions or guidelines, entertaining, or all of the above. We do require them to be educational and/or of scientific interest.*

*Except ECGs, which are captured in the rhythm puzzle section, all images are acceptable, such as echocardiograms, MRI/CT/plain radiographs, angiograms, but also images from measurement equipment used in the clinical setting or at the echolab or cathlab, including measurement graphs (e. g. 24-hour blood pressure measurements, nocturnal oxygen saturation graphs, etc.) Pictures of patients (or a patient’s extremities such as sclerae, abdomen, etc) are accepted when anonymised and with written permission from the patient. Pictures from the coronary care unit or operating theatre are also acceptable.*

*Submit the question and answer as two separate submissions. One figure is accepted in the question (panel 1 and B permitted) and each part must fit on one page.*

## Answer

You will find the answer elsewhere in this issue.

